# Diffuse prothrombotic syndrome after ChAdOx1 nCoV-19 vaccine administration: a case report

**DOI:** 10.1186/s13256-021-03083-y

**Published:** 2021-10-06

**Authors:** Nicole Ceschia, Valentina Scheggi, Anna Maria Gori, Angela Antonietta Rogolino, Francesca Cesari, Betti Giusti, Franco Cipollini, Niccolò Marchionni, Brunetto Alterini, Rossella Marcucci

**Affiliations:** 1 Department of Experimental and Clinical Medicine, University of Florence, Largo G. A. Brambilla 3, 50134 Florence, Italy; 2 Department of Cardiothoracovascular Medicine, AOU Careggi, Largo G. A. Brambilla 3, 50134 Florence, Italy; 3 Department of Internal Medicine, Ospedale San Jacopo, Via Ciliegiole 97, 51100 Pistoia, Italy

**Keywords:** Vaccine-induced immune thrombotic thrombocytopenia, Anti-COVID-19 vaccine, Side effects, Acute limb ischemia, Case report

## Abstract

**Background:**

Vaccine-induced immune thrombotic thrombocytopenia is emerging as one of the most relevant side effects of adenoviral-based vaccines against coronavirus disease 2019. Given the novelty of this disease, the medical community is seeking new evidence and clinical experiences on the management of these patients.

**Case presentation:**

In this article, we describe the case of a 73-year-old Caucasian woman who presented with diffuse prothrombotic syndrome, both in the arterial and venous districts, following the first dose administration of ChAdOx1 CoV-19 vaccine. The main thrombotic sites included the brain, with both a cortical ischemic lesion and thromboses of the left transverse and sigmoid sinuses and the lower limbs, with deep venous thrombosis accompanied by subsegmental pulmonary thromboembolism. The deep venous thrombosis progressively evolved into acute limb ischemia, requiring surgical intervention with thromboendoarterectomy. Anticoagulation was maintained throughout the whole hospitalization period and continued in the outpatient setting using vitamin K antagonists for a recommended period of 6 months.

**Conclusions:**

This case describes the management of vaccine-induced immune thrombotic thrombocytopenia in a complicated clinical scenario, including multisite arterial and venous thromboses. Given the complexity of the patient presentation, this case may implement the comprehension of the mechanisms and clinical features of this disease; it also provides a picture of the challenges related to the management, often requiring a multidisciplinary approach.

## Background

Vaccine-induced immune thrombotic thrombocytopenia (VITT) has recently come to the fore as one of the most relevant and emphasized adverse events related to the administration of adenoviral vector-based anti-coronavirus disease 2019 (COVID-19) vaccines, in particular the ChAdOx1 CoV-19 vaccine (AstraZeneca, University of Oxford, and Serum Institute of India) and the Ad26.COV2.S vaccine (Janssen; Johnson & Johnson) [1]. Initial reports highlighted a newly described syndrome characterized by thrombosis and thrombocytopenia that developed 5–24 days after initial vaccination with ChAdOx1 nCoV-19, with a remarkably high percentage of the patients presenting with thromboses at unusual sites [2–4]. These independent case series showed an apparent predominance of this phenomenon in a young (below 55–60 years of age), female population, even if cases in individuals > 60 years are emerging [5]. In this article, we discuss the case of a 73-year-old woman presenting with diffuse prothrombotic syndrome following the administration of the Vaxzevria vaccine. This case represents a prevalent and overwhelming health problem faced by the medical community, which is still seeking scientific evidence on its diagnosis and treatment options.

## Case presentation

A 73-year-old Caucasian woman presented to the emergency department of a neighboring hospital, complaining of persistent pain in the right popliteal area for few days. The patient admitted she had a sedentary life in the previous period; she was administered the ChAdOx1 CoV-19 vaccine (Vaxzevria) 2 weeks before the clinical presentation. Besides the lower limb pain, the patient presented hemodynamically stable and eupneic, without any neurologic sign or symptom.

Her past medical history revealed only a former appendectomy and a cholecystectomy. As cardiovascular risk factors, she suffered from hypercholesterolemia and arterial hypertension, both under treatment with simvastatin (10 mg), lercanidipine (10 mg), and olmesartan/hydrochlorothiazide (20/25 mg, respectively); she also assumed calcifediol daily.

The patient denied smoking and occasionally consumed alcohol with meals. The only known allergy was to levofloxacin.

Her family history was suggestive for thrombophilia since her mother suffered from thrombophlebitis at a young age and her father died from a not-well-defined thrombosis at age 54 years. She had a brother in apparent good health status.

Given the classical clinical presentation, deep vein thrombosis (DVT) was suspected, and the patient immediately underwent an echo color Doppler (ECD) evaluation of the lower limbs’ veins. The examination confirmed the presence of thrombotic residuals in the right medial gastrocnemius veins.

Therefore, the patient was admitted to a medical ward, where she underwent a complete diagnostic assessment. At presentation, blood tests showed anemia [10.2 g/dl with normal value (n.v.) 12–16 g/dl], thrombocytopenia (nadir value 20 × 1000/mm^3^ with n.v. 130–400 × 1000/mm^3^) and elevated d-dimer concentrations (32,559 μg/ifEU with n.v. < 500 μg/ifEU), while fibrinogen was in the normal range (399 mg/dl, with n.v. 200–400 mg/dl). Considering the high clinical probability of pulmonary embolism (PE), computed tomographic pulmonary angiography (CTPA) was performed. The examination revealed subsegmental thromboembolism in the left inferior pulmonary lobe and, as collateral findings on the abdominal scans, partial thrombosis of the left renal vein and voluminous bilateral adrenal masses (4 cm on the right and 4.5 cm on the left side), compatible with hematomas. Furthermore, cranial computed tomography (CT) displayed a right occipital hypodense lesion extended from the cortex to the ventricle and a small right occipital hyperdensity. Subsequent brain magnetic resonance imaging (MRI) better characterized these findings, confirming the presence of an ischemic lesion in the cortical territory supplied by the right posterior cerebral artery and highlighting thrombosis of the left transverse sinus and sigmoid sinus. Nevertheless, the neurologic examination of the patient remained unremarkable.

Considering the severity of the clinical presentation with multisite thromboses and concomitant thrombocytopenia, in the clinical suspicion of VITT, treatment with high-dose intravenous immune globulin (IVIG) (1 g/kg/day for 2 days) and dexamethasone (40 mg for 4 days) was immediately started. ADAMTS13 activity assays tested negative on repeated determinations (54.8% and 89.4%, with pathological diagnostic value < 30%), excluding the diagnosis of acquired thrombotic thrombocytopenic purpura. Inherited prothrombotic conditions were excluded through the analysis of Leiden factor V G1691A polymorphism, and the factor II G20210A polymorphism; total protein S (93% with n.v. > 70%), free protein S (67% with n.v. > 60% in females), protein S activity (85% with n.v. > 58% in females), and homocysteine levels (20.9 μmol/L with n.v. > 13 μmol/L in females) were in the normal range; anticardiolipin IgM/IgG and anti-β2-GPI IgM/IgG also tested negative (< 20 CU/ml, with the test considered positive if > 20 CU/ml).

The time relation between the symptoms onset and the vaccine administration raised the suspicion of VITT. To confirm the diagnosis, samples for platelet factor 4 (PF4) antibody testing were sent to the core laboratory of Haemostasis and Thrombosis of our Institution. The analysis was performed with two different techniques: enzyme-linked immunosorbent assay (ELISA) tested positive for anti-PF4/hep IgG with 1912 optical density (OD) readings (n.v. OD > 0.400); on the other hand, chemiluminescence immunoassay for anti-PF4/hep IgG gave negative results (0.03 U/ml with n.v. < 1 U/ml). At the first evaluation in a functional assay, the antibodies showed a platelet-activating ability, and after PF4 addition, only in 1/5 donors: the concomitant treatment with high-dose IVIG may explain this result, considering the capability of IVIG to compete with antiPF4 Ab and reduce the platelet activation effect. A subsequent repetition of the test (13 days later) highlighted a substantial reduction in the antibody titer (0.826 OD). According to the available literature [4], these results confirm the diagnosis of VITT.

During the first days of hospitalization, the patient initially developed hypoesthesia with a dorsiflexion deficit of the big toe and fingers on the right foot, which were interpreted as compression symptoms of the right superficial and deep peroneal nerves due to the DVT. On the second day after admission, she suddenly complained of acute pain in the right lower limb with cool extremity, compatible with acute ischemia. At the same time, she developed acute anemia (with a drop of hemoglobin levels from 10.2 g/dL to 7.4 g/dL), without signs of hemolysis, requiring a transfusion with two units of erythrocyte concentrates. This event may be due to retroperitoneal bleeding, considering the presence of adrenal hematomas without signs of hypoadrenalism. An angio-CT of the abdominal and inferior extremity vessels confirmed the occlusion of the right superficial femoral artery, from the distal segment, and of the popliteal artery involving also the posterior and anterior tibial arteries.

Therefore, anticoagulation with fondaparinux was immediately started, and the patient was transferred to our hospital for surgical evaluation. The concomitant thrombocytopenia imposed a low starting dose with fondaparinux 2.5 mg daily, but the subsequent progressive normalization of the platelet count allowed the augmentation of the dosage to full 7.5 mg daily; on the other hand, d-dimer levels remained elevated (about 5000 ng/ml with n.v. < 500 ng/ml) during the whole treatment period. Given the unrelenting symptoms of ischemia corroborated by imaging (with documentation of persistent right femoropopliteal thrombotic occlusion on serial ECD examinations and a repeated angio-CT), the patient subsequently underwent thromboendoarterectomy with Fogarty catheter on the right tibial artery and fasciotomy of the calf.

After the intervention, the patient developed anemia (nadir value 8.1 g/dL with n.v. 12–16 g/dl), requiring blood transfusions, and a progressive decline in the platelet count (nadir value 112 × 10^9^/L with n.v. 140–440 × 10^9^/L). Coagulation assays were also profoundly altered (aPTT 145.6 seconds with n.v. 22–38 seconds, INR 7.5 with n.v. 0.8–1.2); parenteral postoperative anticoagulation was achieved with bivalirudin (dosage 1 mg/kg/hour).

After serial clinical and imaging re-evaluations (through cranial CT) of the cerebral ischemic lesion, excluding its hemorrhagic transformation, fondaparinux treatment (7.5 mg daily) was resumed since the first postoperative day; after 9 days, imbrication with warfarin was started.

The patient showed a slow but progressive improvement in her clinical status, aided by daily wound medications and physical therapy; opiates allowed her to achieve adequate pain control, and steroid therapy was continued throughout the hospitalization. A pre-discharge Doppler evaluation confirmed a normal triphasic flow on the anterior and posterior tibial arteries; a cranial CT, performed after 10 days of effective anticoagulant treatment with warfarin (with INR value between 2 and 3), excluded the presence of expanding intracerebral hemorrhage.

The hospital discharge was on the 17th postoperative day, and the patient was transferred to a rehab facility, already able to move with the help of a walker. She was further re-evaluated at a 1-month follow-up visit, where she presented eupneic and autonomously walking; she denied any bleeding episode, and she was included in the follow-up program for oral antithrombotic therapy of our institution during the following 6 months.

## Discussion

VITT was first described in late February of 2021 as a prothrombotic syndrome that mimics spontaneous heparin-induced thrombocytopenia (HIT) but is triggered by anti-COVID-19 vaccines based on adenoviral vectors. Even if the exact pathophysiologic mechanisms have not yet been completely understood, a major causal role is identified in IgG class antibodies that recognize platelet factor 4 (PF4, also called CXCL4) bound to platelets in a heparin-independent fashion (that is the major difference from HIT) [2]. This binding leads ultimately to platelet activation (and possibly activation of other cells such as neutrophils), resulting in a marked stimulation of the coagulation system and clinically significant thromboembolic complications [1]. As a distinctive feature, thrombosis in VITT occurs both in typical sites of venous thromboembolism, such as PE and DVT in the legs, and in unusual locations, including the splanchnic (splenic, portal, mesenteric) veins, adrenal veins (with risk for adrenal failure if bilateral), and cerebral and ophthalmic veins. Moreover, also arterial thromboses have been described, including ischemic stroke (often, middle cerebral artery) and acute limb ischemia [6]. Therefore, our patient presented the emblematic triad of VITT, with thrombocytopenia, multisite thromboses (both on the arterial—ischemic stroke and acute limb ischemia—and on the venous side—DVT, subsegmental PE, left renal vein, transverse and sigmoid sinuses thromboses), and coagulation abnormalities (elevated d-dimer levels, with fibrinogen in low-normal range). Fortunately, she did not develop the most feared complication of intracranial bleeding, which has been observed in patients with cerebral venous thrombosis while receiving anticoagulation with a heparin product [2, 3], or even spontaneously, probably due to venous congestion.

According to the Italian Society for the Study of Haemostasis and Thrombosis (SISET) position statement [7] and the evolving international recommendations [1], the severity of the thrombocytopenia at presentation (< 50 × 10^9^/L) imposed the immediate initiation of IVIG [8] and dexamethasone, as a means of interrupting VITT antibody-induced platelet activation. Conversely, the absence of worsening intracranial hemorrhage allowed us not to consider platelet transfusion.

The mainstay of VITT treatment is, however, anticoagulation, which is contraindicated only in the case of severe thrombocytopenia (< 20 × 10^9^/L) or worsening intracranial bleeding. Early reports described clinical worsening, even death, in patients treated with heparin [2]. Given the similarity to heparin-induced thrombocytopenia (HIT) and autoimmune HIT (aHIT), experts suggest using a non-heparin anticoagulant, both if an anti-PF4 assay is not available and in the case of anti-PF4 positivity [7]. Accordingly, our patient was treated with fondaparinux (in a body-weight-adjusted dosage) most of the time. Considering the cerebral thrombotic event, prolongation of anticoagulation for 3–6 months has been advised thereafter; warfarin has been the drug of choice, given the absence of randomized controlled trials on direct oral anticoagulants (DOACs) in this context.

## Conclusions

In conclusion, this case gives a comprehensive picture of VITT’s diagnosis and management, displaying a complex clinical scenario, with which clinicians should learn to deal nowadays. The clinical presentation of our patient included many facets of this disease, that together make the therapeutic options more challenging. Hopefully, this case report will improve the comprehension of this emerging pathological condition.

## Data Availability

Not applicable.
